# Implications of Preoperative C-Reactive Protein Levels in Heart Transplant Patients—A Single-Center Retrospective Study

**DOI:** 10.3390/jcm13237466

**Published:** 2024-12-08

**Authors:** Laurentiu Huma, Horatiu Suciu, Calin Avram, Radu-Adrian Suteu, Alina Danilesco, Dragos-Florin Baba, Diana-Andreea Moldovan, Anca-Ileana Sin

**Affiliations:** 1 Department of Cell and Molecular Biology, “George Emil Palade” University of Medicine, Pharmacy, Science and Technology of Târgu Mureș, 540142 Târgu Mureș, Romania; laurentiu.huma@umfst.ro (L.H.); dragos-florin.baba@umfst.ro (D.-F.B.); ileana.sin@umfst.ro (A.-I.S.); 2 Emergency Institute for Cardiovascular Diseases and Transplant, 540136 Târgu Mureș, Romania; horatiu.suciu@umfst.ro (H.S.); radu.suteu@yahoo.com (R.-A.S.); 3 Doctoral School, “George Emil Palade” University of Medicine, Pharmacy, Science and Technology of Târgu Mureș, 540142 Târgu Mureș, Romania; 4 Department of Surgery, “George Emil Palade” University of Medicine, Pharmacy, Science and Technology of Târgu Mureș, 540142 Târgu Mureș, Romania; 5 Department of Medical Informatics and Biostatistics, “George Emil Palade” University of Medicine, Pharmacy, Science and Technology of Târgu Mureș, 540142 Târgu Mureș, Romania; 6 Târgu Mureș County Hospital, 540072 Târgu Mureș, Romania; alinka942@yahoo.com; 7 Department of Family Medicine, “George Emil Palade” University of Medicine, Pharmacy, Science and Technology of Târgu Mureș, 540142 Târgu Mureș, Romania

**Keywords:** heart transplant, C-reactive protein, cardiac resynchronization therapy, inflammation, 6-month mortality

## Abstract

**Background**: Heart transplant is the final therapeutic option for end-stage heart failure patients. It has been used with increasing success as a surgical procedure, greatly influenced by advances in diagnostic and prognostic tools. The aim of this paper was to study potential implications of C-reactive protein (CRP) in patients who underwent heart transplants. **Methods**: Our cohort included 43 adult patients from the Emergency Institute for Cardiovascular Diseases and Transplant of Târgu Mureș who underwent heart transplants in our center between 2011 and 2023. Correlations between CRP levels and different characteristics of the patients were investigated, and the optimal cut-off value for CRP levels in relation to the 6-month mortality rate was determined. The central tendencies of the baseline characteristics of patients who had a CRP value lower than the cut-off and those with a value higher than it were compared using parametric or nonparametric tests. **Results**: Significant correlations between the preoperative CRP levels and 6-month mortality rate (r = 0.35; 95%CI: 0.05–0.60; *p* = 0.02), as well as previous cardiac resynchronization therapy (CRT) and preoperative CRP levels (r = −0.37; 95%CI: −0.61–−0.07, *p* = 0.01) were highlighted. A value for CRP > 1.66 mg/dL was found to be associated with 6-month mortality (OR = 18.00; 95%CI: 1.90–170.33, *p* < 0.01). Moreover, the patients who received CRT before transplantation had significantly lower levels of CRP when compared to those who did not receive CRT (*p* = 0.01). **Conclusions**: Preoperative CRP levels could represent a valuable asset in the follow-up algorithm of heart transplant patients. The lower levels of CRP in patients who benefited from CRT before transplantation highlights the importance of understanding the complex mechanisms of inflammation and increasing focus on device therapy for future transplant recipients. Further prospective studies with larger cohorts are needed for validation.

## 1. Introduction

Advanced heart failure has increased in prevalence over the past few years, becoming a worldwide concern for the medical system, which makes it a focal point for the development of new therapies that could prolong patients’ lifespan and increase their quality of life. Although it is estimated to cover only 0.2–2.8% [1,2] of the total number of heart failure patients, the actual numbers for the advanced stages of cardiac dysfunction and the socio-financial burden they carry require a more rigorous approach in order to identify solutions to known pitfalls [3]. The foremost option of treatment alongside medical therapy in these cases is heart transplantation. The low number of heart transplantation surgeries performed every year is a result of several factors, among which one of the most important is the reduced number of donors [4]. In light of this fact, heart transplantation needs particular consideration in order to achieve positive results regarding survival and low rates of complication in the peri- and post-operative period.

The current survival rate of patients who have undergone heart transplants in excellence centers is around 80–90% at one year, with most deaths occurring in the first few months after the procedure, secondary to acute and subacute complications. This number is higher than those reported in the past (around a 70% survival rate at one year in the 1980s), mostly secondary to the development of new immunosuppressive drugs with more potency and less adverse effects, with an increase in patients’ treatment individualization, as well as better donor and receiver selection [5]

Nowadays, studies attempt to identify factors that could become potential targets in the management and care of these patients. Graft dysfunction and subsequent failure, mainly of the left ventricle, are the most common disclosed causes of poor survival post-surgery [6].

Among the examined factors impacting the long-term course, different immune or inflammatory status markers have been identified as associated predictors of mortality [7]. Besides the widely discussed and explained infectious mechanisms triggering the immune system, the literature also indicates an alleged sterile inflammation that can occur in these patients as a result of innate immune system activation and the perpetuation of these processes [8]. The laboratory detection of an inflammatory process is considerably more accessible compared to the gold standard, endomyocardial biopsy, which has possible complications.

Other studies have highlighted patients’ specific circumstances and status as possible factors that influence the outcome of heart transplantation besides their inflammatory status. Considering the fact that latent forms of cytomegalovirus (CMV) infection can persist in several tissues, such as the myocardium, and they can be reactivated during immunosuppression (as in the case of immunosuppressive treatment after transplants), several studies have highlighted a relationship between CMV infection and post-transplant graft vasculopathy, which may lead to complications and graft rejection [9,10].

Considering the possible short- and mid-term complications, another mechanism focused on during post-surgery medical follow-up is allograft vasculopathy [6]. The background inflammatory process seems to have implications on the development of transplant-related vasculopathy and subsequent allograft failure. The primordial detection of this process is based on baseline C reactive protein (CRP) levels [11]. Nonetheless, the available data on the impact of this biomarker on the prognosis of recipients of heart transplants reflect a contrasting perspective. While values of CRP were observed to have a tendency towards a steady state after a few weeks [12], without a valid correlation with the development of allograft rejection, certain studies have proven an association with the increasing frequency of severe graft rejection during a mean 2-year follow-up period [13].

Therefore, the aim of this paper is to emphasize the role of preoperative CRP in the algorithm for the follow-up of patients with heart transplants and, moreover, to define a cut-off value with significance in a 6-month postoperative clinical evolution. The secondary aim is to investigate potential relationships between different characteristics of the patients and their inflammatory status.

## 2. Materials and Methods

**Figure 1:**
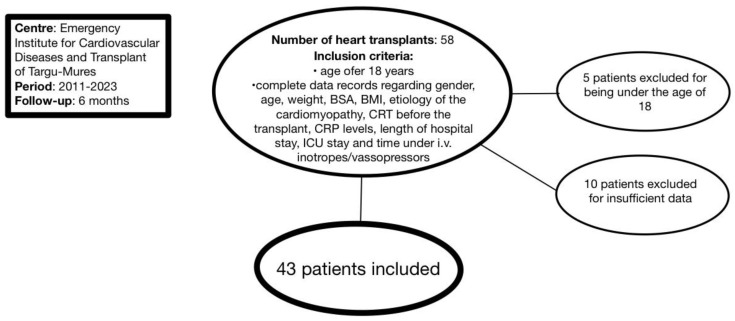
Inclusion criteria. BMI: body mass index; BSA: body surface area; CRP: C-reactive protein; CRT: cardiac resynchronization therapy; ICU: intensive care unit.

Our cohort included adult patients who were admitted to the Emergency Institute for Cardiovascular Diseases and Transplant of Târgu Mureș for heart transplants between 2011 and 2023. The total number of patients who underwent heart transplantation during the selected period was 58, out of which 5 patients were excluded for being under the age of 18, while 10 patients were excluded for incomplete data records. Thus, the final cohort included 43 patients (Figure 1). All patients underwent an orthotopic heart transplant through the bicaval technique, which, in contrast to the biatrial technique, preserves the donor’s right atrium morphology and its conduction system, with patients having a higher reported post-operative chance of maintaining sinus rhythm and lower incidence of mitral and tricuspid regurgitation [14].

Informed consent was obtained from the involved participants. The research was conducted in accordance with the Declaration of Helsinki. The study protocol was approved by the ethics committee of the Emergency Institute of Cardiovascular Diseases and Transplant of Târgu Mureș.

Data collection for baseline characteristics was performed for all patients, including gender, age, weight, body surface area (BSA), body mass index (BMI), etiology of cardiomyopathy, cardiac resynchronization therapy (CRT) before transplantation, length of hospital stay, length of stay in the intensive care unit (ICU), and duration of intravenous (i.v.) inotrope/vasopressor treatment. Regarding CRP levels, values before the transplants were selected and collected from laboratory sheets. Mortality at 6 months was assessed by means of data sheets available in the intranet system of the Emergency Institute for Cardiovascular Diseases and Transplant of Târgu Mures (Figure 1).

**Figure 2:**
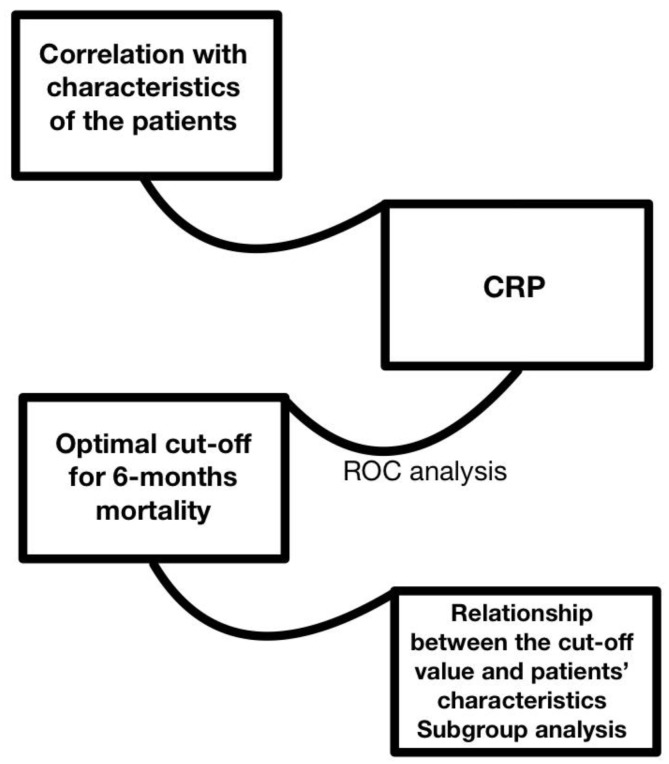
Statistical analysis premise. CRP: C-reactive protein; ROC: receiver operating characteristic.

The second step was to investigate potential correlations between CRP levels and certain parameters of the patients, such as gender, age, weight, BSA, BMI, etiology of cardiomyopathy, CRT before the transplant, duration of hospital stay, ICU stay, and time under i.v. inotropes/vasopressors. This was performed by means of Pearson’s correlation coefficient and Spearman’s rank correlation coefficient, depending on the normality test result for each one of the parameters (Figure 2).

To investigate the significance of CRP values before the transplants, we determined an optimal cut-off value of the inflammatory marker by means of ROC (receiver operating characteristic) analysis and Youden’s index for 6-month mortality. The cut-off value was tested using contingency tables and Fisher’s exact test for a significant association with 6-month mortality. Afterwards, the cohort was divided into two groups, with regard to the patients’ CRP values being over or under the previously determined threshold through ROC analysis. Central tendencies of baseline characteristics in the two newly formed groups were compared using the student’s *t*-test for parametric data and the Mann–Whitney test for nonparametric data. Associations between each one of the groups and dichotomous data of baseline characteristics were investigated by using the Chi-squared and Fisher’s exact tests. Further subgroup analysis was performed using the same methodology (Figure 2).

MedCalc version 22 (MedCalc Software Ltd., Ostend, Belgium) was used to perform the statistical analysis. For quantitative data, maximum, minimum, median, and mean values as well as the standard deviations were determined. Normal distribution of values was assessed using the Shapiro–Wilk (S-W) test. The significance threshold for all the statistical tests was set to 0.05.

## 3. Results

Our cohort was composed of 43 patients who underwent heart transplants between 2011 and 2023 in the Emergency Institute for Cardiovascular Diseases and Transplant of Târgu Mureș. When looking at the baseline characteristics, out of the 43 patients, 86.04% (37:43) were male and 13.96% (6:43) were female. The mean age was 44.46 (SD = 10.45) years, while the mean weight was 76.00 (SD = 15.79) kg. When BSA is considered, a mean value of 1.91 (SD = 0.23) sqm was found, while a mean value of 24.55 (SD = 0.47) kg/sqm characterized the patients’ BMI. Regarding the etiology of the patients’ cardiomyopathy, 74.41% (32:43) had a non-ischemic origin, while 25.59% (11:43) were of ischemic origin. Out of the total number of patients, 20.93% (9:43) underwent CRT (cardiac resynchronization therapy) prior to heart transplantation, while 79.07% (34:43) did not receive this therapy. The mean value for total hospital stay in our cohort was 59.48 (SD = 66.58) days, with a mean ICU (intensive care unit) stay of 51.39 (SD = 63.07) days and a mean duration of i.v. inotrope/vasopressor treatment of 8.74 (SD = 13.20) days (Table 1).

Regarding donor characteristics, 27.90% were female (12:43) and 82.10% were male (31:43). The mean age of the donors was 32.37 (SD = 1.51) years. The mean age difference between the receiver and the donor was 15.34 years. Donor–receiver gender mismatch occurred in 27.90% (12:43) of cases.

Firstly, we investigated potential correlations between the patients’ CRP levels prior to transplantation and their baseline characteristics as well as the 6-month mortality. After applying the S-W test, all calculations were performed using Spearman’s correlation test for nonparametric data. Thus, no statistically significant correlation was found between CRP levels and gender, age, weight, BSA, BMI, etiology of cardiomyopathy, total hospital stay, ICU stay, and time under i.v. inotropes or vasopressors. A significant correlation has been highlighted between the CRP levels and 6-month mortality (*p* = 0.02) and between CRP levels and CRT before transplantation (*p* = 0.01) (Table 2).

**Figure 3:**
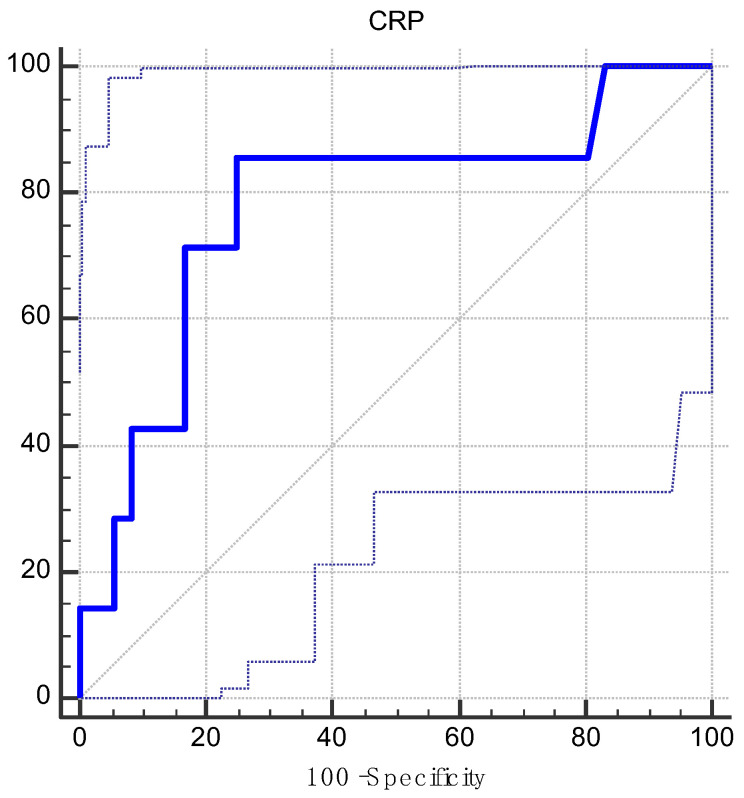
ROC curve for CRP and 6-month mortality. ROC: receiver operating characteristic. CRP: C-reactive protein.

In order to investigate the potential role of CRP as a prognostic factor for mortality at 6 months, we determined the optimal cut-off value of 1.66 mg/dL using an ROC analysis and Youden’s index (Figure 3). This corresponded to an area under the curve (AUC) of 0.78 (CI: 0.62–0.892, *p* = 0.01), with a sensitivity of 85.71% and a specificity of 75.00% (Table 3 and Table 4).

To validate the marker, our cohort was divided into two groups regarding the patients’ CRP values and their relation to the cut-off point. Thus, group 1 (patients with a CRP ≤ 1.66 mg/dL) was composed of 28 patients, while group 2 (patients with a CRP > 1.66 mg/dL) was made up of 15 patients. The two newly formed groups were compared in terms of 6-month mortality using contingency tables and Fisher’s exact test. A significant association between a CRP value before transplantation over 1.66 mg/dL and 6-month mortality was found (OR = 18.00; 95%CI: 1.90–170.33, *p* < 0.01). Central tendencies for age, weight, BSA, BMI, total hospital stay, ICU stay, and time under i.v. inotropes or vasopressors between group 1 and 2 were compared using corresponding parametric or nonparametric tests after performing the S-W test. No statistically significant differences between central tendencies in the two groups were found for age, weight, BSA, BMI, length of hospital stay, ICU stay, or time under i.v. inotropes or vasopressors. To investigate potential associations between CRP values lower than the cut-off point and gender, etiology of cardiomyopathy, and CRT before transplantation, we used contingency tables and the Chi^2^ test with Yates’s correction, as well as Fisher’s exact test. We found no significant association between gender and a CRP value under 1.66 mg/dL, nor between the etiology of cardiomyopathy and a CRP value under the cut-off point. After using Fisher’s exact test to determine a potential association between CRT prior to transplantation and CRP values lower than the cut-off point, no statistically significant result was obtained (Table 5).

Lastly, we performed an analysis of the initial cohort divided into two new groups regarding CRT. A total of nine patients had received CRT before transplantation, while 34 patients did not receive CRT. The CRT group had a mean (SD)/median CRP value of 0.41 (0.28)/0.35 mg/dL, while the non-CRT group was characterized by a mean (SD)/median CRP value of 2.45 (2.54)/1.50 mg/dL. To investigate any statistically significant differences in the central tendency for CRP values between the two groups, we used the Mann–Whitney test for nonparametric data with *p* = 0.01.

## 4. Discussion

Considering the pivotal role of heart transplants in end-stage heart failure and the challenges faced in the selection and management of heart transplant recipients [15], there is a need for the development of new predictive markers for outcomes and for broadening the usage of existing ones. Thus, thorough investigations of available resources for further clinical value are an important part of modern research.

Inflammation plays a central role in the management and follow-up of patients with transplants. Associations between inflammatory markers and complications such as cardiac allograft vasculopathy have been identified [16], while therapeutic interventions that may lower the extent of inflammation are an important research field [17].

CRP value and derivates have been found to have a predictive role in several diseases and procedures, including major abdominal surgery [18], gastric cancer [19], renal transplantation [20], and COVID-19 infection [21]. The high versatility of this marker is proof of its value. Thus, CRP levels before transplantation were the focal point of our study, with an emphasis on associations and correlations with different characteristics and outcomes of patients with heart transplants.

We included adult patients with end-stage heart failure who underwent heart transplantation and were admitted to the Emergency Institute for Cardiovascular Diseases and Transplant of Targu-Mures between 2011 and 2023. After excluding underage patients and those with incomplete data records, a final cohort of 43 patients was formed. The 6-month survival rate in our center was 83.73%, similar to other European centers [5].

Firstly, we investigated a potential link between CRP levels before transplantation and the baseline characteristics of the patients. Correlations between lower CRP levels and CRT have been found, as well as lower CRP levels and 6-month survival.

CRT has been shown to influence CRP levels in heart failure patients, increasing the benefits of the therapy itself by limiting inflammatory systemic processes. This effect has been highlighted both through a reduction in inflammatory markers [22,23,24] and a reduction in the expression of inflammation-promoting genes [25].

Cardiac resynchronization therapy is one of the more recent pillars of heart failure management, having proven its usefulness in treating patients with dilated cardiomyopathies, decreased systolic function, symptomatic heart failure, and intraventricular conduction abnormalities. As patients who are scheduled for heart transplantation suffer from end-stage heart failure, most of these individuals also develop dilated cardiomyopathy, as a primary cause of heart failure or as a consequence of other diseases (e.g., ischemic heart disease). The left-ventricular dilation associated with decreased systolic function is also the foundation for intraventricular conduction disease, since the majority of patients with dilated cardiomyopathy involving the left chambers also develop left bundle branch block(LBBB). Physiologically, the first ventricular segment that goes through depolarization and then contraction is the base segment of the interventricular septum. In contrast, the last segment to depolarize is the postero-lateral wall of the left ventricle. In normal circumstances, due to the His–Purkinje system, the entire left-ventricular wall reaches maximum contraction before the closing of the aortic valve, thus ejecting the maximum amount of oxygenated blood into systemic circulation. When intraventricular conduction disease has developed, as is the case in the presence of LBBB, there is a consequent delay in impulse conduction; thus, the ventricular segments that were to contract last are further delayed. As a direct consequence, they will not reach full contraction before the closing of the aortic valve, and less blood will be ejected, with an impact on the ejection fraction and systolic function. Cardiac resynchronization therapy is performed by the implantation of a cardiac pacemaker with three sensing pacing leads in the right atrium, right ventricle, and in a tributary branch of the coronary sinus, as close to the postero-lateral wall of the left ventricle as possible. Using the sensing and pacing properties of the leads, clinicians set the pacemaker to follow the best algorithm for each patient in order to reduce inter-, intra-, and atrioventricular dyssynchronism [26,27,28,29,30].

The second step in our study was to determine an optimal cut-off value for preoperative CRP levels in order to investigate the relationship between this marker and 6-month mortality as well as other characteristics of the patients. Thus, using ROC analysis and Youden’s index, we determined a cut-off value for CRP at 1.66 mg/dL, which corresponded to a sensitivity of 85.71% and a specificity of 75.00%. Afterwards, our cohort was divided into two groups regarding their relation to the cut-off value for CRP, and a comparison of group characteristics was made. We found a significant association between a preoperative CRP value of >1.66 mg/dL and 6-month mortality, which suggests the importance of preoperative inflammatory status to the outcome of patients with heart transplant. There was also an association between preoperative CRT and preoperative CRP levels of ≤1.66 mg/dL, which proved to be very close to a level of statistical significance, suggesting a close relationship between CRT and preoperative inflammatory status.

To further investigate a potential relationship between CRT and CRP values in our cohort, we performed a second division of the initial cohort of 43 patients with regard to the presence or absence of a CRT device prior to transplantation and compared the medians of the CRP levels between the groups. Thus, we found a statistically significant difference between preoperative values of CRP between the patients who benefited from CRT before heart transplantation and those who did not undergo this procedure. This finding highlights the complexity of the effects that implantable devices can have on systemic processes such as inflammation and the strong relationship between device therapy and patients’ outcomes.

The main cause of death at the 6-month threshold in our cohort was represented by advanced heart failure, which ultimately led to multiple system organ failure (MSOF) and death. The patients’ statuses were altered either during their initial admission or during their secondary admission for worsening symptoms of heart failure.

By further validating the findings of our study, an important pillar of the therapeutic management of patients who are scheduled for a heart transplant can be developed. CRP is a low-cost, widely available laboratory parameter that can be measured in most hospitals. In practice, the validation of a threshold for CRP over which mortality and complications have a higher chance of occurring would inform the physician’s approach to the patient, as a more thorough investigation of the causes for CRP increases should be performed, potentially involving supplementary members to the transplant team (e.g., a rheumatologist). Furthermore, the validation of a threshold would have an impact on the preparation process for transplantation, as therapies that lower CRP (e.g., statins) [31] might be considered even when the current guideline criteria for their initiation is not met.

Alongside circulatory biomarkers, several different prognostic parameters have been tested in heart transplantation, with results that can open the way for a different approach to postoperative management. Notably, imaging markers have made important progress in recent years, with several studies highlighting the importance of different parameters in patients with heart transplants [32,33,34,35,36,37].

Studying both invasive and noninvasive markers is of utmost importance for the development of complex prediction scores, which could influence the management of patients by combining information derived from clinical and paraclinical tests [36,37,38,39,40]. The combination of echocardiographic markers, genetic testing, and biomarkers has proven useful in diagnosing rare diseases that might become the starting point of heart failure as a disease [41]. In the case of early detection, such pathologies can become a target for treatment, thus delaying the development of end-stage heart failure.

From another point of view, the introduction of novel markers and prognostic scores that may influence management and patient follow-up may not only influence the quality of life and overall health of patients, but those of their close relatives as well. Considering the psychological burden that the care of end-stage heart failure patients imposes on their families [42,43], better selection and management may also improve both the physical and mental health of heart transplant recipients and their families.

## 5. Limitations

In the circumstances of rapidly developing diagnostic and prognostic techniques that may influence the management algorithms of patients with heart transplants, the limitations of our study have to be mentioned. The retrospective design of the study may be a source of error, secondary to differential losses to follow-up, information bias, and the absence of data on potential confounding factors (e.g., differences in the management of associated diseases between referring centers and between patients from the first to last years of the research) [44]. The lack of legislation on the default status of individuals as organ donors and need for written informed consent from a close relative of a potential organ donor, which must be obtained under pressing circumstances in a state of grief, are reasons for the relatively low pool of donors in Romania. The lack of informational campaigns through mass media channels on the benefits and potential life-saving implications of organ donation has also contributed to the scarcity of organs [4,45]. The small cohort may also influence the results through false-positive or -negative data, which may become a ground for misinterpreting the relationships between events, confidence intervals, and statistical significance [44]. To further evaluate the relationship between preoperative CRP levels, CRT, and mortality, prospective studies with larger cohorts are required.

Additional logistic limitations of this study are a consequence of the region-specific reluctance of individuals to enroll as organ donors. The low number of heart transplants resulted in a wider time interval during which data were collected. This has a series of consequences, the most important one being the possible differences in heart failure management between the first datasheet analyzed in the study (2011) and the last (2023), considering the updated versions of the European Society of Cardiology Guidelines (dated from 2021) and the introduction of SGLT2 (sodium-glucose cotransporter-2) inhibitors. Furthermore, differences in the protocol applied to heart transplant patients, especially in the early years of the study, had an impact on the collection of different laboratory parameters, as the protocols did not include the same parameters in all datasheets, thus limiting the inclusion of more biomarkers in the study. Moreover, the doses of immunosuppressive drugs received by the patients had a large degree of individual variability, in accordance with the histopathological results of the cardiac biopsies and clinical parameters, with their adverse effects being a possible contributor to the subsequent evolution of the patients. Another point of interest to be studied in the future by enrolling patients in a larger, prospective study is the point in time at which patients received a pacemaker for cardiac resynchronization therapy, as patients who are enrolled later in a study that extends over a few years might receive better therapies, which could influence their prognosis, irrespective of the presence or absence of CRT.

While the relationship between CRP levels and acute events are well established and the latter should be assessed and treated before a heart transplant, there has been less emphasis on the modifiable organic characteristics of patients scheduled for heart transplantation, with the main reason being the intensity of symptoms, which increases the difficulty of making lifestyle changes. Medications which decrease inflammation (e.g., statins, NSAIDs, and steroids) will also consequently decrease CRP levels, thus making the development of an algorithm which takes into account variables other than the baseline characteristics of patients necessary for validating cut-off values of inflammatory markers. It should also be noted that CRP values can be influenced by modifiable risk factors, such as smoking, physical activity, diet, or BMI, further highlighting the necessity of the clinical individualization of algorithms and including cut-off values in decision-making scores rather than using them by themselves [46].

## 6. Conclusions

We investigated potential correlations between preoperative CRP values and different baseline characteristics of heart transplant patients in our center, as well as the 6-month mortality rate. Significant correlations between inflammatory status and mortality and between cardiac resynchronization therapy and CRP values have been highlighted. Afterwards, we determined the optimal cut-off value of >1.66 mg/dL for CRP and divided the cohort into two groups with regard to their relation to the cut-off point. A significant association was found between a CRP value over the cut-off and the 6-month mortality rate, while the association of CRT and CRP values ≤ 1.66 mg/dL was close to the level of statistical significance (*p* = 0.06). Considering the closeness of the latter association to the level of significance, we further investigated this relationship by dividing the initial cohort into two groups regarding the presence or absence of CRT before transplantation. Significant differences in CRP median values have been identified between the two groups.

## Figures and Tables

**Table 1:** Baseline characteristics.

Parameter	Result
Gender, males (n, %)	37 (86.04)
Age, years (Mean, SD)	44.46 (10.45)
Weight, kg (Mean, SD)	76.00 (15.79)
BSA, sqm (Mean, SD)	1.91 (0.23)
BMI, kg/sqm (Mean, SD)	24.55 (4.07)
Etiology of cardiomyopathy, non-ischemic (n, %)	32 (74.41)
CRT before the transplant, yes (n, %)	9 (20.93)
Total hospital stay, days (Mean, SD)	59.48 (66.58)
ICU stay, days (Mean, SD)	51.39 (63.07)
Time under i.v. inotropes/vasopressors, days (Mean, SD)	8.74 (13.20)

BMI: body mass index; BSA: body surface area; CRT: cardiac resynchronization therapy; ICU: intensive care unit; SD: standard deviation.

**Table 2:** Correlations of the CRP levels.

Parameter	r (95% CI)	*p* Value
Males	0.03 (−0.27–0.34)	0.81 *
Age	0.04 (−0.27–0.34)	0.78 *
Weight	0.12 (−0.18–0.41)	0.40 *
BSA	0.16 (−0.15–0.45)	0.29 *
BMI	0.12 (−0.19–0.41)	0.41 *
Nonischemic cardiomyopathy	−0.01 (−0.32–0.29)	0.92 *
CRT	−0.37 (−0.61–−0.07)	**0.01** *
Hospital stay	<0.01 (−0.31–0.30)	0.99 *
ICU stay	0.09 (−0.22–0.39)	0.54 *
Time under i.v. inotropes or vasopressors	−0.03 (−0.33–0.28)	0.84 *
6-month mortality	0.35 (0.05–0.60)	**0.02** *

* Spearman’s rank correlation. BMI: body mass index; BSA: body surface area; CRP: C-reactive protein; CRT: cardiac resynchronization therapy; ICU: intensive care unit.

**Table 3:** Area under the curve (AUC) for CRP.

AUC	0.78
95% CI	0.62–0.89
z statistic	2.50
*p* value	0.01

**Table 4:** Maximum Youden index.

Youden index	0.60
Associated criterion	**>1.66**
Sensitivity	85.71
Specificity	75.00

**Table 5:** Analysis of the two groups.

Parameter	Group 1	Group 2	*p* Value
6-month mortality (n, %)	1 (3.57)	6 (40.00)	**<0.01** ^Ψ^
Gender, male (n, %)	23 (82.14)	14 (93.33)	0.40 ^Ψ^
Age, years, Mean (SD)/Median	43.32 (11.08)/43.50	46.60 (9.13)/46.00	0.33 *
Weight, kg, Mean (SD)/Median	74.64 (17.41)/73.50	78.53 (12.36)/78.00	0.44 *
BSA, sqm, Mean (SD)/Median	1.89 (0.25)/1.88	1.96 (0.17)/1.99	0.36 *
BMI, kg/sqm, Mean (SD)/Median	24.26 (4.39)/24.10	25.08 (3.47)/24.60	0.63 **
Etiology of cardiomyopathy, nonischemic (n, %)	21 (75.00)	11 (73.33)	1.00 ^Φ^
CRT before transplantation, yes (n, %)	9 (32.14)	0 (0.00)	0.06 ^Ψ^
Hospital stay, days, Mean (SD)/Median	50.53(30.99)/40.00	79.57(107.93)/36.50	0.86 **
ICU stay, days, Mean (SD)/Median	40.75 (23.95)/33.50	71.26 (100.88)/34.00	0.49 **
Time under i.v. inotropes/vasopressors, days, Mean (SD)/Median	5.60 (3.97)/4.00	14.60 (20.88)/5.00	0.89 **

* *t*-test assuming equal variances, ** Mann–Whitney, ^Ψ^ Fisher’s exact test, ^Φ^ Chi^2^ test with Yates’s correction.

## Data Availability

All the data generated or analyzed during this study are included in this published article.
